# Association between social capital and utilization of essential public health services among elderly migrants: a multilevel logistic study based on the 2017 China migrant dynamic survey (CMDS)

**DOI:** 10.1186/s12889-024-18726-0

**Published:** 2024-05-14

**Authors:** Qi Luo, Xiaolei Chen, Linlin Zhao, Qinghua Hu, Juan Du, Shuang Shao

**Affiliations:** School of General Practice and Continuing Education, Capital Medical University, No.10 Xitoutiao, You An Men, Beijing, 100069 China

**Keywords:** Elderly migrants, Social capital, Essential public health services, Multilevel logistic regression analysis

## Abstract

**Background:**

As the number of elderly migrants in China continues to grow, it is necessary to pay closer attention to their health and health services. Some studies have confirmed that social capital plays a significant role in the utilization of health services. Therefore, an in-depth exploration of the relationship between social capital and the utilization of essential public health services (EPHS) by elderly migrants will not only contribute to improving their overall health but also facilitate a more balanced development of public health service system in China.

**Methods:**

Based on the cross-sectional data from the 2017 China Migrants Dynamic Survey (CMDS), this study examined the impact of social capital on the utilization of EPHS among elderly migrants. We evaluated social capital at two distinct levels: the individual and the community, and considered two dimensions of social capital: structural social capital (SSC) and cognitive social capital (CSC). The study aimed to delve into the impact of these forms of social capital on the utilization of EPHS among elderly migrants, and whether the migration range moderates this impact by multilevel logistic regression analysis.

**Results:**

A total of 5,728 migrant elderly individuals were selected. The health records establishment rate and health education acceptance rate were approximately 33.0% and 58.6%, respectively. Social capital influenceed the utilization of EPHS among elderly migrants. Specifically, individual-level SSC and CSC have impacts on both the establishment of health records (OR = 1.598, 95%CI 1.366–1.869; OR = 1.705, 95%CI 1.433–2.028) and the acceptance of health education (OR = 1.345, 95%CI 1.154–1.567; OR = 2.297, 95%CI 1.906–2.768) among elderly migrants, while community-level SSC only affected the acceptance of health education (OR = 3.838, 95%CI 1.328–11.097). There were significant differences in individual-level SSC, health records, and health education among different migration range subgroups among elderly migrants. Migration range moderated the effect of social capital on the utilization of EPHS, crossing provinces could weaken the relationship between SSC and health education.

**Conclusions:**

Social capital is associated with a higher utilization rate of EPHS among elderly migrants. It is necessary to encourage them to actively participate in social activities, strengthen public services and infrastructure construction in the area, and improve their sense of belonging and identity.

**Supplementary Information:**

The online version contains supplementary material available at 10.1186/s12889-024-18726-0.

## Introduction

With the booming of China’s economy and the development of urbanization, migrant population has increased significantly. The proportion of elderly migrants is continually on the rise in the context of an ageing population and family migration [1]. As of 2015, there were over 13 million elderly migrants, accounting for 5.3% of the total million migrants, with an increase of 8 million and an annual growth rate of 6.6% during the past 15 years  [2]. According to the " China Migrant Population Development Report 2018”, the trend of increasing elderly migrants has persisted since 2015 [3]. The dual challenges of “migration” and “ageing” are particularly evident among elderly migrants. Age-associated decline in health and the deterioration of bodily functions, with a corresponding increase in demand for health services [4, 5]. Previous research indicated that 22.0% of elderly migrants suffered from chronic disease in China [6]. Furthermore, they often face difficulties of adapting to new environments, accessing social support, and receiving medical services, due to their displacement from familiar surroundings and shrinking social networks, that impact their overall health status ultimately [4, 7]. Therefore, the Chinese government has taken a series of measures to promote their health status.

    In 2009, the Chinese government launched the EPHS program, which is considered a core mission of the public health system [3]. This program provided free health services to all residents, especially to key populations such as children, pregnant women, the elderly, and individuals with chronic diseases [8]. Currently, EPHS in China encompasses 14 components, including health records, health education, and free health screenings, etc [9]. However, it showed that the utilization rate of these services among elderly migrants remains low in 2017, due to they faced many barriers to access EPHS in the destination areas [10]. Specifically, the establishment rate of health records is only 38.0%, the coverage rate of chronic disease health education is only 39.0%, and the participation rate in free health examinations is only 45.9% [11, 12], which significantly lower than the rates of the general population and fall short of the national targets set [13]. It indicated that further efforts are needed to promote the utilization of EPHS among elderly migrants.

The seeking behavior for health services, including public health services, clinical prevention, and medical care [14], is influenced by a range of complex factors such as demographic characteristics (e.g., gender, age, marital status), socioeconomic factors (e.g., education level, income), individual health status, and social environmental factors (e.g., cultural background, policy environment) [15–18]. Some scholars believe that the utilization of EPHS is a complex process involving the joint efforts of multiple entities, including government leadership, the participation of grassroots public health personnel, and the recognition of community residents. The improvement of the utilization effect of EPHS not only depends on government policy incentives and the fulfillment of EPHS functions, but also relies on the active participation, trust, and collaboration of residents [19]. Social capital can well cover these factors and provide new ideas for improving the utilization rate of EPHS.

The concept of social capital was proposed officially by Bourdieu, which believed that it is related to the size of an individual’s social network and the amount of capital contained [20]. The definition of social capital was adopted widely in the field of health from Putnam and colleagues, which defined as characteristics of social organizations from a collective perspective, such as trust, norms, and social networks, to promote cooperation and enhance social efficiency [21]. Moreover, Kawachi viewed social capital as resources obtained by individuals and groups through their social networks at the individual and community levels [22]. Based on the interpretation of social capital above, social capital of this study refers to the resources that individuals or collectives obtain through their social connections, which can promote reciprocal cooperation and collective action within society.

In order to gain a deeper understanding of the nature and mechanism of social capital, studies often classify social capital into different dimensions, whether at the individual or community level [23]. For example, a common classification divides social capital into cognitive and structural types. Cognitive social capital (CSC) refers to the intangible part of social capital, including norms, values, attitudes and beliefs, which is measured by people’s perception of interpersonal trust and reciprocity. Structural social capital (SSC) refers to the observable part of a social organization, such as the density of social networks or the pattern of citizen participation, which is usually measured by the degree of participation in various forms of social activities [24]. Some scholars divide social capital into bonding, bridging, and linking dimensions. Bonding social capital mainly exists within closely connected homogeneous groups, bridging social capital exists between heterogeneous groups, and linking social capital, also known as vertical social capital, refers to the relationship between horizontal organization and different classes [25–27]. Considering the database had measures relating to citizen participation and social belonging, this classification of SSC and CSC is adopted in the study.

Recently, some researchers have tried to explore the complex relationship between social capital and the utilization of EPHS among migrants. The Chinese study has revealed that social capital has a positive impact on the utilization of EPHS among migrants aged 15 to 59 years [28]. However, the relationship between different dimensions of social capital and the utilization of EPHS among migrants is inconsistent. Comparing with individual-level social capital, community-level social capital has a more significant effect on the utilization of EPHS [29]. SSC had a greater impact on the establishment of health records and the receipt of health education than CSC [29]. A study also found that SSC had positive effects on physical examination and management of chronic disease among elderly migrants [30], but CSC had a positive or negative impact on healthcare utilization. If group norms promote the use of health services, healthcare utilization will increase; if group norms discourage the use of health services, healthcare utilization will decrease [31–33].

The impact of social capital on the utilization of EPHS among the migrant population is depended not only on the dimensions of social capital but also on various factors such as cultural background, economic status, and the focus of study [34]. For instance, researchers suggest that migrating to eastern regions or across provinces may hinder access to health services [35, 36], as these factors can influence the integration of the migrants into new environments and the accumulation of social capital. Meanwhile, the cultural, economic backgrounds, and social norms align with their host city, migrants can accumulate social capital more easily. Moreover, unlike young migrants, most elderly individuals have withdrawn from the labor market in their new locations due to migrate for caring their children [37, 38], and local governments may be reluctant to bring them into localized management systems [39]. Although some studies have confirmed the positive impact of social capital on the utilization of EPHS among young migrants, it remains uncertain whether a similar relationship exists among elderly migrants.

Therefore, in order to enrich empirical evidence on the association between social capital and the utilization of EPHS, based on data from the 2017 China Migrant Dynamics Survey (CMDS), this study explored the following issues: Firstly, the characteristics of social capital distribution and the utilization of EPHS among subgroups of elderly migrants across different migration ranges; Secondly, the relationship between different dimensions of social capital and the utilization of EPHS among elderly migrants; Finally, how migration range influences the relationship between social capital and the utilization of EPHS among elderly migrants. This study provides a theoretical foundation for promoting the utilization level of EPHS among elderly migrants, which is crucial for achieving healthy and active aging, building a healthy China, and effectively responding to public health challenges.

## Methods

### Study design and population

The data was obtained from the 2017 wave of the China Migrant Dynamic Survey (CMDS). The CMDS is a nationally representative cross-sectional survey of migrants conducted annually by the National Health Commission of the People’s Republic of China. It covers 31 provinces and the Xinjiang Production and Construction Corps in China. Respondents were aged above 15, and the purpose of going out was mainly to live and work, excluding travel, medical treatment, business trips, family visits, etc. The survey targeted the non-local population (without household registration) who lived in the local area for one month or more. The research adopted a stratified multi-stage random sampling method with the Probability Proportional to Size (PPS) sampling method. Specifically, towns and villages were first selected using the PPS method from 32 units, including provinces and districts. Then, villages or community residents’ committees were selected using the PPS sampling method. Subsequently, local officials of each village provided information about all members of 50 families or 100 migrants to the sampling platform, and 20 migrants were randomly chosen from different families. With 95% confidence, the error was controlled within 3%, and a total of 169,989 people were surveyed.

    This study adopted individual questionnaire A, which covers five aspects: individual sociodemographic characteristics, migrant status, housing and employment status, health status and the utilization status of EPHS, and the state of social integration. The study focused on migrants aged 60 and over who had resided in the area for more than six months. The final sample comprised 5,728 individuals. All participants provided informed written consent prior to participating in the face-to-face interview. The raw data from the 2017 CMDS used in this study was approved by the National Health Commission of the People’s Republic of China. Data and details are available on the website https://www.chinaldrk.org.cn/wjw/#/user/apply.

### Measures

#### Dependent variables

Health records and health education were selected as indicators to measure the utilization of EPHS. The utilization of health records was assessed by the question “Have you established a local health record?” and the answer was “Yes” or “No”. Health education was assessed by the question “Have you received any of the following health education in your community in the past year?”. Respondents could choose from nine options, including occupational disease prevention and control, tuberculosis prevention and control, chronic disease prevention and control, sexually transmitted diseases and AIDS prevention and control, tobacco control, self-help education in public emergencies, mental health education, etc. Individuals who did not receive any category of above-mentioned health education were marked as “No”, and those who received one or more categories of above health education were marked as “Yes”.

### Social capital

The independent variable is social capital. Based on prior research [29], we assessed two aspects: individual-level social capital and community-level social capital, specifically civic participation and social belonging. Civic participation is regarded as SSC, and social belonging is reflected in CCS [40]. Community-level data were aggregated by province districts.

SSC refers to the presence of formal opportunity structures or activities in which individuals build or strengthen their social connections (like social networks and civic participation) [40]. This study selected civic participation as SSC. Civic participation was dichotomized into (1= “Yes” vs. 0= “No”), responses assessed by the five questions: “Since 2016, have you made suggestions to your unit/community/village or supervised the unit/community/village affairs management”, “Since 2016, have you participated in property donation, blood donation, volunteer activities, etc.”, “Since 2016, have you reported the situation/put forward policy suggestions to relevant government departments in various ways “, “Since 2016, have you posted online comments on national affairs and social events or participated in related discussions”, “Since 2016, have you participated in party/youth league organization activities and party branch meetings”. Each question responds to four levels: “no”, “occasionally”, “sometimes”, and “frequently”. It was scored 0 if the respondent chose “no” for all the above items, or scored 1 if the respondent chose “other answers” for any of the above questions.

CSC generally refers to individuals’ perceptions, beliefs, and attitudes toward their social surroundings (like trust, social belonging in cities and reciprocity between neighborhoods) [40]. This study selected social belonging as CSC. Social belonging was assessed by the two levels of “lower” or “higher” from four questions: “I like the city/place living now”, “I am very willing to blend with the local people and become a part of them”, “I think the local people are willing to accept me as a part of them”, and “I think I am one of the local people”, each of the four questions was assigned between 1 and 4 points(response scale: “totally disagree”, “disagree”, “agree”, “in full agree”), then was divided into two levels by using the median of total points.

We conducted a factor analysis using a total of 9 questions, including five items about civic participation and four questions about social belonging. We used factor scores aggregation as community-level social capital scores in each province district. Before aggregation, we calculated the ${R}_{wg}$, ${ICC}_{1}$, and ${ICC}_{2}$(Table 1), which indicated that individual-level social capital can aggregated better.

### Control variables

We used individual sociodemographic characteristics, health status, and community-level characteristics as control variables.

Individual-level factors included gender, age (60–64, 65–69, 70–74, or ≥ 75), marital status (single or currently married), education (primary school or below, junior or senior high school, and undergraduate or above), family monthly income (was divided into four quartiles, Q1to Q4, with Q1 and Q4 indicating the lowest and the highest incomes), reason of migrant (work for business, take care of others, provide for the aged), migration range(across cities within province, across provinces), time to medical facility (below 15 min, 15 min or above), self-rated health (poor or good). Community-level variables included the number of primary medical institutions in 2016 (a continuous variable).

### Statistical analysis

Descriptive statistics were used to summarize characteristic variables related to individual and community-level social capital, and utilization of EPHS. Subsequently, we compared differences in individual-level social capital, health education, and health records among different migration range groups of migrants using cross-tabulation and chi-square tests. Next, we used multilevel logistic regression to assess the effect of social capital on the utilization of EPHS. The data for this study comprised individuals (first level) nested within province districts (second level). The multilevel analysis framework assumes that individual outcomes are partially dependent on the province districts. First, we ran a null model of multilevel logistic regression the intra-class correlation coefficients for health records and health education were 6.16% and 14.57%, respectively, indicating variation across provincial districts. This necessitated the use of multilevel logistic regression. In model 1, we added control variables like gender, age, marital status, education, family monthly income, reason for migrant, time to medical facility, self-rated health, migration range, and community-level variables. In model 2, we examined the association between individual-level social capital, community-level social capital, and the utilization of EPHS. In model 3, we included interaction terms of social capital with migration range into the model to analyze the moderating effect of migration range on the correlation between social capital, health education, and health records.

Descriptive statistics was conducted using SPSS 25.0, and multilevel logistic regression was conducted using R software (V.3.6.3 for Windows). Significance was set at *p* < 0.05.

## Results

### Characteristics of elderly migrants

Table 2 shows the characteristics of 5,728 elderly migrants. The mean age of them was 66.0 years (SD = 5.6); 57.6% were male. 84.0% were married; 48.3% of elderly migrants received an education level of primary school or below; and the average monthly family income was 6,001.6 RMB (SD = 6,739.3); 34.5% were migrated for working; 75.3% lived less than 15 min away from medical facility; and the number of primary medical institutions in peer province averaged 26,994.8(SD = 19,969.1). Overall, 80.6% of elderly migrants thought they were healthy.

### Social capital and the utilization of EPHS among elderly migrants

Table 3 indicates that 33.0% of elderly migrants established health records, and 58.6% had received at least one category of health education. In terms of individual-level social capital, only 28.7% participated in civic activities, and 54.0% believed they had high social belonging.

### Comparison of social capital and utilization of EPHS among different subgroups

According to Table 4, the level of civic participation among elderly migrants from different provinces was higher than that among elderly migrants from different cities within the same province. However, the number of elderly migrants from different provinces who had established health records and received health education was significantly lower than that of elderly migrants from different cities within the same province.

### Associations between social capital and utilization of EPHS among elderly migrants

As shown in Table 5, we analyzed the association between social capital and the utilization of health records. In Model 3, after considering the hierarchical structure of the data and adjusting for control variables, we found a correlation between social capital and the establishment of health records. Higher individual-level SSC and CSC were significantly associated with higher rates of health record establishment (OR = 1.598, 95% CI 1.366 to 1.869; OR = 1.705, 95% CI 1.433 to 2.028). However, the interaction effects between migration range and community-level SSC and CSC, individual-level SSC and CSC were not significant.

Table 6 takes the same set of analyses to explore the relationship between social capital and the utilization of health education. It shows that higher levels of community-level SSC, individual-level SSC and CSC are significantly associated with higher rates of acceptance of health education (OR = 3.838, 95% CI 1.328 to 11.097; OR = 1.345, 95% CI 1.154 to 1.567; OR = 2.297, 95% CI 1.906 to 2.768). Among the interaction terms between migration range and social capital, the OR value for migration range and community-level SSC was significantly less than 1, while the interactions between migration range and community-level SSC and CSC, and individual-level CSC were not significant.

**Table 1:** Results of *R*_*wg*_ and ICC

Community-level social capital	*N*	*R*_*wg*_		*ICC*_1_	*ICC*_2_
		Mean (MD)	Minimum, maximum		
Civic participation	31	0.96(0.96)	0.90–0.99	0.10	0.84
Social belonging	31	0.90(0.90)	0.86–0.93	0.15	0.88

**Table 2:** Elderly migrants’ characteristics(*N* = 5,728)

Variables	Subgroups	Overall	Health Records		Health Education	
			No(*N*/%)	Yes(*N*/%)	No(*N*/%)	Yes(*N*/%)
Control Variables						
Gender	Male	3,302(57.6)	2,234(58.2)	1,068(56.4)	1,309(55.2)	1,993(59.4)
	Female	2,426(42.4)	1,602(41.8)	824(43.6)	1,063(44.8)	1,363(40.6)
Age(years)	60–64	2,903(50.7)	2,020(52.7)	883(46.7)	1,179(49.7)	1,724(51.4)
	65–69	1,559(27.2)	1,020(26.6)	539(28.5)	640(27.0)	919(27.4)
	70–74	727(12.7)	463(12.1)	264(14.0)	313(13.2)	414(12.3)
	75 or older	539(9.4)	333(8.7)	206(10.9)	240(10.1)	299(8.9)
Marital status	Single	915(16.0)	642(16.7)	273(14.4)	429(18.1)	486(14.5)
	Married	4,813(84.0)	3,194(83.3)	1,619(85.6)	1,943(81.9)	2,870(85.5)
Education	Primary school or below	2,767(48.3)	1,867(48.7)	900(47.6)	1,212(51.1)	1,555(46.3)
	Junior or senior high school	2,590(45.2)	1,709(44.6)	881(46.6)	1,009(42.5)	1,581(47.1)
	Undergraduate or above	371(6.5)	260(6.8)	111(5.9)	151(6.4)	220(6.6)
Family monthly income(yuan)	Quartile1	1,383(24.1)	930(24.2)	453(23.9)	633(26.7)	750(22.3)
	Quartile2	1,475(25.8)	932(24.3)	543(28.7)	588(24.8)	887(26.4)
	Quartile3	1,368(23.9)	885(23.1)	483(25.5)	528(22.3)	840(25.0)
	Quartile4	1,502(26.2)	1,089(28.4)	413(21.8)	623(26.3)	879(26.2)
Reason of migrant	Work for business	1,979(34.5)	1,381(36.0)	598(31.6)	746(31.5)	1,233(36.7)
	Take care of children	1,910(33.3)	1,264(33.0)	646(34.1)	849(35.8)	1,061(31.6)
	Provide for the aged	1,723(30.1)	1,117(29.1)	606(32.0)	735(31.0)	988(29.4)
	Others	116(2.0)	74(1.9)	42(2.2)	42(1.8)	74(2.2)
Migration range	Across cities within province	3,208(56.0)	1,232(65.1)	1,976(51.5)	1,259(53.1)	1,949(58.1)
	Across provinces	2,520(44.0)	660(34.9)	1,860(48.5)	1,113(46.9)	1,407(41.9)
Time to medical facility, minutes	15 or below	4,313(75.3)	2,812(73.3)	1,501(79.3)	1,755(74.0)	2,558(76.2)
	15 or above	1,415(24.7)	1,024(26.7)	391(20.7)	617(26.0)	798(23.8)
The number of primary medical and health institutions		26,994.8 ± 19,969.1				
Health status						
Self-rated health	No	1,110(19.4)	720(18.8)	390(20.6)	527(22.2)	583(17.4)
	Yes	4,618(80.6)	3,116(81.2)	1,502(79.4)	1,845(77.8)	2,773(82.6)

**Table 3:** Social capital and the utilization of EPHS of elderly migrants

Variables		*N*(%)
Individual-level social capital		
SSC	No	4,084(71.3)
	Yes	1,644(28.7)
CSC	Low	2,634(46.0)
	High	3,094(54.0)
Community-level social capital		Minimum, maximum(mean)
SSC		-0.45-10.57(0)
CSC		-5.12-1.24(0)
EPHS		
Health Records	No	3,836(67.0)
	Yes	1,892(33.0)
Health education	No	2,372(41.4)
	Yes	3,356(58.6)

**Table 4:** A cross-table showing the effect of migration range on social capital and the utilization of EPHS

Variables	Subgroups	Migration range differences		X^2^
		Across cities within province	Across provinces	
SSC	No	2,351(73.3)	1,733(68.8)	
	Yes	857(26.7)	787(31.2)	14.064***
CSC	Low	1,450(45.2)	1,184(47.0)	
	High	1,758(54.8)	1,336(53.0)	1.810
Health Records	No	1,976(61.6)	1,860(73.8)	
	Yes	1,232(38.4)	660(26.2)	95.174***
Health education	No	1,259(39.2)	1,113(44.2)	
	Yes	1,949(60.8)	1,407(55.8)	14.087***

Note: ***p < 0.001, **p < 0.01, *p < 0.05

**Table 5:** Multilevel logistic regression on the effects of social capital and health records

Variables	Model 1	Model 2	Model 3
	OR (95%CI)	OR (95%CI)	OR (95%CI)
Gender (ref. Male)			
Female	1.217(1.069,1.385) **	1.220(1.070,1.391) **	1.224(1.074,1.396) **
Age (ref. 60–64 years)			
65–69	1.275(1.107,1.470) ***	1.273(1.103,1.469) ***	1.271(1.101,1.467) **
70–74	1.310(1.084,1.582) **	1.306(1.078,1.581) **	1.307(1.079,1.583) **
≥ 75	1.343(1.081,1.669) **	1.368(1.099,1.703) **	1.374(1.103,1.710) **
Marital status (ref. Single)			
Married	1.294(1.091,1.535) **	1.253(1.054,1.489) *	1.262(1.061,1.500) **
Education (ref. Primary school and below)			
Junior or senior high school	1.254(1.098,1.432) ***	1.174(1.026,1.343) *	1.180(1.032,1.351) *
Undergraduate or above	1.514(1.149,1.995) **	1.246(0.940,1.653)	1.257(0.947,1.667)
Family monthly income (ref. Quartile1)			
Quartile2	1.341(1.136,1.584) ***	1.353(1.143,1.601) ***	1.354(1.144,1.603) ***
Quartile3	1.289(1.077,1.542) **	1.266(1.055,1.518) *	1.260(1.050,1.511) *
Quartile4	1.138(0.936,1.382)	1.096(0.900,1.335)	1.094(0.898,1.333)
Reason of migrant (ref. Work for business)			
Take care of children	1.154(0.996,1.337)	1.164(1.003,1.351) *	1.168(1.006,1.355) *
Provide for the aged	1.382(1.135,1.683) **	1.374(1.126,1.676) **	1.374(1.126,1.677) **
Others	1.084(0.708,1.660)	1.033(0.670,1.592)	1.032(0.670,1.592)
Time to medical facility (ref. 15 or below)			
15 or above	0.692(0.601,0.798) ***	0.697(0.604,0.805) ***	0.697(0.604,0.805) ***
Self-rated health (ref. No)			
Yes	1.026(0.881,1.194)	1.012(0.867,1.180)	1.008(0.864,1.176)
Number of primary medical and health institutions	1.060(0.933,1.204)	1.065(0.939,1.208)	1.053(0.932,1.191)
Migration range (ref. Across cities within province)			
Across provinces	0.899(0.779,1.037)	0.916(0.792,1.058)	0.934(0.748,1.167)
Community-level SSC (ref. Low)			
High		0.430(0.090,2.047)	0.626(0.130,3.018)
Community-level CSC (ref. Low)			
High		1.790(0.536,5.979)	1.705(0.516,5.631)
SSC (ref. No)			
Yes		1.661(1.451,1.902) ***	1.598(1.366,1.869) ***
CSC (ref. Low)			
High		1.550(1.370,1.753) ***	1.705(1.433,2.028) ***
Community-level SSC* Migration range			0.917(0.712,1.181)
Community-level CSC* Migration range			0.944(0.720,1.237)
SSC* Migration range			0.400(0.136,1.174)
CSC* Migration range			1.261(0.653,2.435)
-2LL	3331.272	3272.963	3271.006

Note: ***p < 0.001, **p < 0.01, *p < 0.05

**Table 6:** Multilevel logistic regression on the effects of social capital and health education

Variables	Model 1	Model 2	Model 3
	OR (95%CI)	OR (95%CI)	OR (95%CI)
Gender (ref. Male)			
Female	1.009(0.895,1.137)	1.012(0.896,1.142)	1.015(0.899,1.146)
Age (ref. 60–64 years)			
65–69	1.051(0.921,1.199)	1.042(0.911,1.191)	1.041(0.910,1.190)
70–74	0.986(0.826,1.177)	0.986(0.824,1.180)	0.987(0.825,1.182)
≥ 75	0.984(0.802,1.207)	0.995(0.808,1.224)	0.999(0.812,1.230)
Marital status (ref. Single)			
Married	1.253(1.074,1.461) **	1.213(1.038,1.418) *	1.221(1.045,1.428) *
Education (ref. Primary school and below)			
Junior or senior high school	1.285(1.135,1.454) ***	1.172(1.033,1.330) *	1.180(1.040,1.338) **
Undergraduate or above	1.361(1.063,1.742) **	1.035(0.801,1.336)	1.049(0.812,1.354)
Family monthly income (ref. Quartile1)			
Quartile2	1.196(1.022,1.399) *	1.186(1.012,1.391) *	1.186(1.011,1.391) *
Quartile3	1.219(1.030,1.443) *	1.169(0.985,1.387)	1.162(0.979,1.380)
Quartile4	1.098(0.919,1.311)	1.026(0.856,1.230)	1.025(0.855,1.229)
Reason of migrant (ref. work for business)			
Take care of children	0.784(0.684,0.899) ***	0.794(0.691,0.913) **	0.798(0.694,0.917) **
Provide for the aged	0.824(0.681,0.996) *	0.810(0.667,0.982) *	0.809(0.667,0.982) *
Others	0.917(0.609,1.379)	0.874(0.578,1.324)	0.875(0.577,1.326)
Time to medical facility (ref. 15 or below)			
15 or above	0.926(0.815,1.052)	0.927(0.814,1.055)	0.927(0.814,1.055)
Self-rated health (ref. No)			
Yes	1.273(1.104,1.468) ***	1.243(1.076,1.437) **	1.239(1.072,1.432) **
Number of primary medical and health institutions	1.005(0.923,1.093)	1.018(0.939,1.104)	1.006(0.929,1.088)
Migration range (ref. Across cities within province)			
Across provinces	0.952(0.831,1.090)	0.956(0.833,1.097)	0.962(0.791,1.169)
Community-level SSC (ref. Low)			
High		2.414(0.890,6.543)	3.838(1.328,11.097) *
Community-level CSC (ref. Low)			
High		0.920(0.424,1.997)	0.836(0.380,1.836)
SSC (ref. No)			
Yes		2.189(1.914,2.503) ***	1.345(1.154,1.567) ***
CSC (ref. Low)			
High		1.304(1.164,1.461) ***	2.297(1.906,2.768) ***
Community-level SSC* Migration range			0.927(0.737,1.165)
Community-level CSC* Migration range			0.913(0.701,1.189)
SSC* Migration range			0.340(0.125,0.925) *
CSC* Migration range			1.425(0.774,2.622)
-2LL	3,730.488	3,643.226	3,639.995

Note: ***p < 0.001, **p < 0.01, *p < 0.05

## Discussion

Elderly migrants were considered as a special target group in the process of promoting the equalization of EPHS in China. Overall, the level of social capital among elderly migrants is relatively low, whether it is SSC or CSC. However, compared with CSC (social belonging), their level of SSC (civic participation) is even lower, similar with the findings from studies on the general migrant population [29, 41]. This study also revealed significant differences in the utilization of EPHS and SSC among the elderly with varying mobility ranges. Specifically, elderly individuals who migrate across provinces were less likely to establish health records and receive health education, while engaged in more social interactions, that aligned with previous research findings [42]. A survey focused on elderly migrants in Shenzhen and Guiyang found that it is more challenging for those who migrate across provinces to access relevant support resources [42]. They tended to actively interact with others and participate in social activities, to foster new interpersonal relationships as a means to accumulate social capital and gain access to welfare protections in their new locations [37, 42, 43].

Social capital has a positive impact on the utilization of EPHS, as confirmed by previous studies, such as its ability to enhance the utilization rates of preventive health care services and family doctor services [44, 45]. This is because social interactions among elderly individuals enable them to acquire information related to health service utilization and raise their awareness of health and access to health service [45, 46]. Additionally, civic engagement and a sense of social belonging can strengthen the trust of elderly individuals in local community health services and among residents [47, 48]. Based on this trust, they are more receptive to information about service utilization, fostering shared health beliefs and promoting the establishment of common health behavior norms, thus effectively increasing their service utilization rates [46].

The impact of CSC on the utilization of EPHS is greater than that of SSC, which is consistent with the previous research findings [49, 50]. The establishment of health records and access to health education rely more on the individual willingness. In communities with strong senses of belonging and trust, older adults can receive more social support and have higher awareness of participation in activities [51, 52]. A study in Japan indicated that the elderly with mobility disability in communities with high levels of trust could obtain more assistance, including hitchhiking and home services [51], in turn facilitating their access to health services. Furthermore, some studies indicated that CSC can foster SSC [53]. In communities with a high cohesion and social belonging, more activities will be hold, including health education, free medical consultations, to makes the elderly easier to utilize relevant resources. Meanwhile, regional policy support can partially substitute for SSC, and it plays a complementary role with CSC [50].

The results of this study indicate that community-level SSC has a positive impact on the receipt of health education among elderly migrants. However, previous researches on the effects of community-level social capital on health service utilization have yielded mixed findings. Some studies reported a positive association [44, 54], and others found no significant relationship between these factors [55]. The varying impacts of community-level social capital on health service utilization may stem from differences in how scholars define the concept of “community”. Some studies define community at the neighborhood or school district level, which is more susceptible to individual-level factors. Since many local organizations and activities occur within these boundaries, it may be easier to detect a correlation between community-level social capital and health service utilization. On the other hand, some studies define community at the state or provincial level, which is influenced by cultural and economic policies [33, 34]. In such cases, it can be more challenging to discern the relationship between community-level social capital and health service utilization. Therefore, when conducting research, it is important to clearly define the concept of “community” and consider the influencing factors at different geographical levels. This approach can help to more accurately reveal the relationship between community-level social capital and health service utilization.

Cross-provincial migration can weaken the impact of civic engagement on the receiving of health education. Some research findings indicated that elderly individuals who migrate across provinces were less likely to utilize health services, that might be related to the policy and institutional factors [56]. In China, community health service centers could not grasp the information of migrants timely and accurately, due to no establishment of a comprehensive cross-regional electronic health records transfer and sharing system. It is difficult for elderly migrants who migrate across provinces to access health education information [57], in turn, diminish the impact of civic engagement on the utilization of receiving health education among elderly migrants. Moreover, it is likely that elderly individuals migrate across provinces in especial will face more cultural background differences and social exclusion, resulting in feelings of helplessness and loneliness in the process of establishing social connections with other. Diminishing social interactions can also undermine access to health education for elderly migrants.

Our study has some limitations. Firstly, although social capital is a multidimensional concept, our research only focused on the dimensions of civic participation and social belonging, due to the limitations of the database. Future research is necessary to design or utilize questionnaires that comprehensively cover various dimensions of social capital to understand the relationship between social capital and EPHS among elderly migrants. Secondly, considering the limited sample size of smaller units (such as cities, communities, etc.) in the database, provinces were selected as the community unit, which may be too large for the community level. Thirdly, as this is a cross sectional study, we can not determine a causal relationship between social capital and utilization of EPHS. Instrumental variables can then be used for analysis, or prospective longitudinal studies can be conducted to elucidate the causal relationships and mechanisms between social capital and EPHS utilization among elderly migrants. Lastly, this study also simplified the indicators of EPHS, which may bias the interpretation of results.

## Conclusions

This study found that higher social capital was associated with higher utilization of EPHS among elderly migrants. It is suggested that government sectors should promote the utilization of EPHS among elderly migrants by developing interventions targeting social capital.

First, improving SSC among elderly migrants. Community service agencies can organize health education and social activities to promote their active participation, which can increase communication opportunities with the local population. Second, increasing CSC. The government can enhance elderly migrants’ sense of security and belonging of community, through the establishment of a friendly community (improving the community infrastructure, optimizing the living environment and providing free event spaces). Finally, establishing a reliable information access of EPHS. Community health service institutions should establish information platforms such as network push and short note prompts, to disseminate the information about EPHS.

### Electronic Supplementary Material

Below is the link to the electronic supplementary material.

Supplementary Table 1: Factor analysis suitability test.
Supplementary Table 2: Explanation of the total variance of social capital and factor contribution rate.

## Data Availability

National Health Commission of the People’s Republic of China. 2022. “China Migrant Dynamic Survey (CMDS).”. https://www.chinaldrk.org.cn/wjw/#/user/apply.
