# Supplementary material for: Association between social capital and utilization of essential public health services among elderly migrants: a multilevel logistic study based on the 2017 China migrant dynamic survey (CMDS)
Source: BMC Public Health. 2024 May 14;24:1252. doi: 10.1186/s12889-024-18726-0 (PMC11092042; doi:10.1186/s12889-024-18726-0)
Supplement: Supplementary file 1 — Supplementary Table 1: Factor analysis suitability test. Supplementary Table 2: Explanation of the total variance of social capital and factor contribution rate. [file 12889_2024_18726_MOESM1_ESM.docx]

**Supplementary Table 1** Factor analysis suitability test

| Number of questions | KMO value | Bartlett Spherical test |
| --- | --- | --- |
| 9 | 0.777 | <0.001 |

**Supplementary Table 2** Explanation of the total variance of social capital and factor contribution rate

| Factor | Initial eigenvalues | | | Rotation squares and loads | | |
| --- | --- | --- | --- | --- | --- | --- |
|  | Summary | Variance（%） | Cumulative variance（%） | Summary | Variance（%） | Cumulative variance（%） |
| Factor1 | 2.853 | 31.699 | 31.699 | 2.776 | 30.844 | 30.844 |
| Factor2 | 1.997 | 22.192 | 53.891 | 2.074 | 23.047 | 53.891 |
